# MAp34 Regulates the Non-specific Cell Immunity of Monocytes/Macrophages and Inhibits the Lectin Pathway of Complement Activation in a Teleost Fish

**DOI:** 10.3389/fimmu.2020.01706

**Published:** 2020-08-12

**Authors:** Liangliang Mu, Xiaoxue Yin, Hairong Wu, Kailiang Han, Zheng Guo, Jianmin Ye

**Affiliations:** Guangdong Provincial Key Laboratory for Healthy and Safe Aquaculture, School of Life Sciences, Institute of Modern Aquaculture Science and Engineering, South China Normal University, Guangzhou, China

**Keywords:** *Oreochromis niloticus*, MAp34, non-specific cell immunity, competitive inhibition, lectin pathway

## Abstract

The lectin pathway of the complement system is one of the main components of innate immunity, which plays a pivotal role in the defense against infectious microorganisms and maintains immune homeostasis. However, its control mechanisms remain unclear in teleost fish. In this study, we described the identification and functional characterization of a mannose-binding lectin associated protein MAp34 (OnMAp34) from Nile tilapia (*Oreochromis niloticus*) at molecular, cellular, and protein levels. The open reading frame (ORF) of *OnMAp34* is 918 bp of nucleotide sequence encoding a polypeptide of 305 amino acids. The deduced amino acid sequence has three characteristic structures, including two C1r/C1s-Uegf-BMP domains (CUB) and one epidermal growth factor domain (EGF). Expression analysis revealed that the *OnMAp34* was highly expressed in the liver and widely existed in other examined tissues. In addition, the mRNA and protein expression levels of OnMAp34 were remarkably altered upon infection with *Streptococcus agalactiae* and *Aeromonas hydrophila in vivo* and *in vitro*. Further, we found that the OnMAp34 could participate in the non-specific cellular immune defense, including the regulation of inflammation, migration, and enhancement of phagocytosis of monocytes/macrophages. Moreover, the OnMAp34 could compete with OnMASPs to combine OnMBL and inhibit the lectin pathway of complement activation. Overall, our results provide new insights into the understanding of MAp34 as a potent regulator in the lectin complement pathway and non-specific cell immunity in an early vertebrate.

## Introduction

Complement is the central part of innate immunity that plays a key role in the defense against pathogens and homeostasis (1). The complement system can be activated depending on the context by three different pathways, including the classical pathway (CP), the alternative pathway (AP), and the lectin pathway (LP), of which the LP serves as the first line of defense against pathogen invasion (2, 3). The LP is initiated when mannose-binding lectin (MBL) or ficolin (FCN) binds appropriate carbohydrate or acetylated patterns of microorganism and forms complexes with MBL-associated serine proteases (MASPs) (4, 5). Then, the complement system is activated by a series of enzymatic cascades, such as the cleavage of complement factors C4 and C2, the cleavage and deposition of the central complement component C3 into C3b, the formation of the C5 convertase, and the recruitment of C6, C7, C8, and C9 (2, 3, 6, 7). The result of the complement activation eventually leads to lysis and killing of pathogenic microorganisms through the membrane attack complex (MAC) (2, 7).

MASPs belong to the serine-protease superfamily and contain five regulatory domains (CUB1-EGF-CUB2-CCP1-CCP2) and a serine protease domain (SP), which play indispensable roles in the control of complement activation by LP (7, 8). In mammals, members of the MASP family have been reported to include three serine proteases, MASP-1, MASP-2, and MASP-3, and two non-enzymatic proteins, MAp44 (MAp-1) and MAp19 (sMAP) (5, 9). Among them, MASP-1, MASP-3, and MAp44 are splicing variants from the *MASP1* gene, while MASP-2, and MAp19 are splicing variants from the *MASP2* gene (5, 10). In general, MASP-2 is capable of cleaving C4 and C2 to form C3 convertase, and further downstream activation leads to cleavage and deposition of C3b (11, 12). Recent studies show that MASP-2 activation strictly depends on MASP-1, which produces 60% of C2a responsible for the C3 convertase formation (7, 13). Therefore, MASP-1 and MASP-2 may be indispensable for the LP of complement activation.

The LP is the key to inducing immune response and plays a vital role in the host resistance to pathogen infection. However, uncontrolled activation of complement can lead to excessive inflammation, causing tissue damage and even necrosis or autoimmune disease, requiring control mechanisms (14, 15). The non-enzymatic MAp44 is an alternative splicing product of MASP-1 and only contains the two CUBs, the EGF, and the first CCP domain, which can function as a potent inhibitor of the LP activation *in vivo* (5, 16, 17). Similarly, a truncated protein of MASP-2, MAP19 contains only one CUB and EGF domain, which may act as a regulator of the LP activation by competitively binding MBL and FCN (18, 19). Further, C1 inhibitor (C1INH), an inhibitor for C1r and C1s, forms equimolar complexes with MASPs and prevents proteolytic activity, which plays a vital regulation role in the CP and LP (20, 21). Apart from the function in complement activation, MASPs also play other multi-functional functions (7). MASP-1 can play roles in a variety of biological effects, including coagulation, enzymatic hydrolysis of factor XIII and fibrinogen, cell activation, and inflammation regulation (7, 22, 23). Moreover, MAp44 also participates in the regulation of inflammatory reaction and has been considered to be a potential available therapy of inflammatory arthritis (24, 25).

In teleosts, the structure and function of MASPs have been investigated in a few species. In common carp (*Cyprinus carpio*), MASP-2 was cloned and analyzed, which could participate in the catalytic activation of C4 (26). MASP-1 could mediate immune responses of host resistance to *A. hydrophila* invasion from grass carp (*Ctenopharyngodon idella*) (27). In addition, we have presented the identification of the OnMBL, OnMASP-1, and OnMAp44 from Nile tilapia (*O. niloticus*), which exert pivotal roles in the host defense of innate immunity (25, 28, 29). Interestingly, a novel MBL-associated protein (MAp34) from *Cynoglossus semilaevis* was identified, which was found to possess hemolytic and bacteriostatic activity and regulation of immune cell activity (30). However, there are few studies on the related immune mechanism, especially in the regulation of the LP and non-specific cell immunity.

In the present study, we reported the expression and function of a mannose-binding lectin/ficolin-associated protein from *O. niloticus*, which was denominated *OnMAp34*. The full length of *OnMAp34* was identified and analyzed. The expression profiles of OnMAp34 were explored in different tissues and cells upon pathogen infection (*S. agalactiae* and *A. hydrophila*). Further, the effect of recombinant OnMAp34 protein on non-specific cell immunity including inflammation, migration, and phagocytosis, were demonstrated. Moreover, we found that OnMAp34 could compete with OnMASPs to combine OnMBL and inhibit the lectin pathway of complement activation. Therefore, our results provide new insights into the understanding of MAp34 as an important regulator in the lectin complement pathway and non-specific cell immunity in an early vertebrate.

## Materials and Methods

### Animals

Nile tilapia, about 80 ± 10 g (for challenge experiment) and 300 ± 20 g (for cell separation) were procured from the Guangdong Tilapia Breeding Farm (Guangzhou, China), which were cultured as previously described (28, 31). The Balb/c female mice for the preparation of polyclonal antibody (pAb) were purchased from Guangdong Medical Laboratory Animal Center (Guangzhou, China). All animal protocols were reviewed and approved by the University Animal Care and Use Committee of the South China Normal University.

### Immunization and Sample Collection

To characterize the tissue distribution of *OnMAp34*, the samples from healthy *O. niloticus* were collected, including liver, head kidney, hind kidney, spleen, skin, gills, peripheral blood, thymus, muscle, and intestine. To explore the variation of *OnMAp34* after bacterial challenges, the experimental fish were intraperitoneally injected with 0.1 mL live *S. agalactiae* (ZQ0910, 1 × 10^7^ CFU/mL) or *A. hydrophila* (BYK00810, 1 × 10^7^ CFU/mL) (28, 31). The control group was stimulated with 0.1 mL sterilized phosphate-buffered saline (PBS, 10 mM phosphate, 150 mM NaCl, pH 7.4) per fish. Liver, spleen, and head kidney samples were collected at time points of 0, 3, 6, 12, 24 h, 2, 3, 5, and 7 d post-immunization. All of the collected samples were immediately frozen in liquid nitrogen and deposited at −80°C.

### Cloning and Analysis of *OnMAp34*

The full-length sequence of *OnMAp34* was identified based on the predicted sequence of *O. niloticus MAp34* (GenBank accession XM_031726339.1). All of the primers in this study were summarized in Table 1. The sequence analysis referred to the previous description (28). A phylogenetic tree was constructed with molecular evolution genetics analysis (MEGA) software version 6.0, and 1,000 bootstrap replications were applied to evaluate reliability (32).

**Table 1:** Primers used in this study.

**Primers**	**Nucleotide sequence (5^**′**^-3^**′**^)**	**Purpose**
MAp34-F	CCTCTATGTTGTTGCTCTTTTTTCT	Full cDNA
MAp34-R	CAGTCTGAAGTCAAAGTAAGGGTCT	Full cDNA
M13-F	TGTAAAACGACGGCCAGT	Sequencing
M13-R	CAGGAAACAGCTATGACC	Sequencing
EMAp34-F	CGCGGATCCGTGGAAATGACAGGGTTATACGGC	Protein expression
EMAp34-R	CGCAAGCTTAGTCTGAAGTCAAAGTAAGGGTCT	Protein expression
β-actin-F	CGAGAGGGAAATCGTGCGTGACA	Control
β-actin-R	AGGAAGGAAGGCTGGAAGAGGGC	Control
qMAp34-F	CCAGGTTTTGGCAGAGGGG	qRT-PCR
qMAp34-R	GTTTCGGGCTGACAGGATGA	qRT-PCR
qIL-1β-F	CGTGCCAACAGTGAGAAAGCG	qRT-PCR
qIL-1β-R	CAGGAGGGACGGAAGGGAT	qRT-PCR
qIL-6-F	ACAGAGGAGGCGGAGATG	qRT-PCR
qIL-6-R	GCAGTGCTTCGGGATAGAG	qRT-PCR
qTNF-α-F	GCTGAGGCTCCTGGACAAAA	qRT-PCR
qTNF-α-R	TCTGCCATTCCACTGAGGTCTT	qRT-PCR
qIL-10-F	TGGAGGGCTTCCCCGTCAG	qRT-PCR
qIL-10-R	CTGTCGGCAGAACCGTGTCC	qRT-PCR
qIL-8-F	GATAAGCAACAGAATCATTGTCAGC	qRT-PCR
qIL-8-R	CCTCGCAGTGGGAGTTGG	qRT-PCR
qCCL3-F	AGACCACTCACATCCTTTTGCTG	qRT-PCR
qCCL3-R	GCAGGCTTTGGGCATCG	qRT-PCR
qMCP-1BF	TGTCCTTCATCACCCATACGC	qRT-PCR
qMCP-1BR	TGCTGCCTTCAGTGTTGTGG	qRT-PCR
qMIF-F	CACATCAACCCTGACCAAAT	qRT-PCR
qMIF-R	GCCTGTTGGCAGCACC	qRT-PCR

### Quantitative Real-Time PCR

Total RNAs and cDNA from abovementioned samples were extracted and synthesized using a Trizol Reagent and PrimerScript™ RT reagent kit with a gDNA Eraser (TaKaRa, Japan) as in previous descriptions (28, 31). The quality and concentration of RNA were determined by the Nanodrop 2000 assay (Thermo Fisher, USA). qRT-PCR was performed through the 7500 Real Time PCR System (Life Technologies, USA) with SYBR premix ExTaq™ II (Takara, Japan). The reaction volume and program were the same as before (28, 31). β-actin was used as an internal control to normalize the relative expression levels of *OnMAp34* (28, 31, 33). Fold changes of *OnMAp34* expression level were normalized against β-actin by the 2^−ΔΔCt^ method (34).

### Isolation of Hepatocytes and Monocytes/Macrophages

The preparation of primary *O. niloticus* hepatocytes was performed as in previous descriptions (31, 35–37). Briefly, the livers were dissected and digested with 0.1% trypsin (0.2% EDTA) (Sigma, USA) for 20 min. After washing, the cells were re-suspended with the addition of a 3-mL red blood cell lysis buffer (KangWei, China) on the ice for 3 min. Then the cells were washed and adjusted to 1 × 10^6^ cells/mL within the DMEM medium.

Monocytes/macrophages (MO/MΦ) were separated from the head kidney as in previous descriptions (33, 37–39). Briefly, the cells were isolated through a 54%/31% discontinuous percoll (Sigma, USA) density gradient and centrifuged at 500 × g at 4°C for 40 min. The cells were collected and adjusted to 1 × 10^7^ cells/mL with the L-15 medium (Gibico, USA), then finally cultured at least 24 h at 25°C. After removing the non-adherent cells, the MO/MΦ were re-suspended and adjusted to 1 × 10^6^ cells/mL with the L-15 medium.

The experimental group was stimulated with formalin-inactivated *S. agalactiae* or *A. hydrophila* (1 × 10^7^ CFU/mL), and the control group was only stimulated with sterile 1 × PBS. All the groups were maintained at 25°C, and cells were collected at the time points of 0, 3, 6, 12, 24, and 48 h post-stimulation.

### Expression and Purification of OnMA34

The ORF of *OnMAp34* was amplified by the specific primers with restriction sites (*BamH I* and *Hind III*) EMAp34-F and EMAp34-R (Table 1). The expression plasmid Trx-pET-32a-MAp34 was constructed and transferred into *E. coli* BL21 (DE3) (TianGen, China). Then the cells were cultured in LB-ampicillin at 37°C until O.D. 600 reached 0.6–0.8. Subsequently, the bacteria were induced by 1 mM isopropyl-β-D-thiogalactopyranpside (IPTG) for 5 h at 37°C. After ultrasonic (Xin Zhi, China) crushing, the recombinant protein was purified with His Band Resin columns (Novagen, Germany) according to the protocol and analyzed by a 12% SDS-PAGE. Moreover, the Trx-pET-32a (Trx) was also prepared for subsequent experiments (28).

### Preparation of Mouse Polyclonal Antibodies

The (r)OnMAp34 was used as an antigen to immunize mice for preparation pAb as described previously (28). After the third immunization, the titer of pAb reached a level of 512,000 units/mL, then the serum was collected and stored at −80°C in a refrigerator. The specificity of the pAb to OnMAp34 was detected by Western blot.

### Immunofluorescence Identification of OnMAp34

To explore the protein expression levels of OnMAp34 in the liver and spleen upon pathogen infection, the immunofluorescence was performed according to previous descriptions (28). The primary antibody was the pAb of (r)OnMAp34; the fluorescent antibody was goat-anti-mouse IgG Alexa 488 (Thermo, USA). The immunofluorescence was detected by a fluorescence microscope (Leica, Germany).

### ELISA to Determine OnMAp34 Concentration

The OnMAp34 concentration in the Nile tilapia serum and cell supernatant was tested by a competitive inhibition ELISA assay as reported previously (25, 40). Briefly, the 96-well plates (Corning, USA) were coated with (r)OnMAp34 (2 μg/mL), diluted with a coating buffer (0.05 M carbonate-bicarbonate buffer, pH 9.6) at 4°C overnight. The plates were then blocked with 0.5% BSA-TTBS for 2 h at 37°C. The stimulated serum (2-fold dilution), culture supernatant, and mouse anti-OnMAp34 pAb (1:3,200, the optimal dilution determined previously) were placed in each well and incubated for 1 h. The second antibody was goat anti-mouse IgG antibody (1:2,000) (Southern Biotech, USA). Finally, the result was detected by a microplate reader (Thermo, USA) at O.D. 405 and calculated from pre-made standard curves.

### Expression Analysis of Inflammatory Cytokines and Chemokines

In order to investigate the (r)OnMAp34 (endotoxin removal) playing an important role in inflammation and migration response, the experiment was implemented as in previous descriptions (25, 29). Briefly, the expressions of interleukin-1β (*IL-1*β), interleukin-6 (*IL-6*), tumor necrosis factor α (*TNF-*α), interleukin-10 (*IL-10*), chemokine 8 (*CXCL-8*), chemokine ligand 3 (*CCL3*), monocyte chemotactic protein 1B (*MCP-1B*), and macrophage migration inhibitory factor (*MIF*) were tested in MO/MΦ. The MO/MΦ was stimulated with (r)OnMAp34 (5 μg/mL), Trx (5 μg/mL), and 1× PBS as a control. Cells were collected at different time points for subsequent analysis by qRT-PCR. Moreover, the Trx protein (endotoxin removal) was prepared as reported previously (28).

### Assessment of Phagocytosis

To explore the effect of (r)OnMAp34 on phagocytosis, flow cytometer (FCM) analysis was implemented as previous descriptions (33, 37). Briefly, the MO/MΦ (1 × 10^6^ cells/mL) phagocytosing the FITC-labeled *S. agalactiae* and *A. hydrophila* (1 × 10^7^ CFU/mL) were analyzed by FCM (BD, USA), in which the bacteria were pre-incubated with 100 μL of TBS, Trx (100 μg/mL), or (r)OnMAp34 (100 μg/mL) for 1 h. The non-ingested bacteria were removed, and the cell suspension was centrifuged over a cushion of 3% BSA in TBS supplemented with 4.5% D-glucose at 100 × g at 4°C for 10 min, repeated three times; then the pellets were re-suspended with TBS, and 1 μL of ice-cold trypan blue (0.4%) was added per sample (20). The 10,000 individual cells were analyzed in each sample. Data analyses were implemented by Flowjo X.

### Binding of (r)OnMAp34 to OnMBL

The interaction of OnMAp34 and OnMBL was performed by ELISA. Briefly, OnMBL (2 μg/mL) was incubated with increasing concentrations (0, 1, 2, 5, and 10 μg/mL) of (r)OnMAp34, (r)OnMAp44, or (r)OnMASP-1, respectively. The Trx and (r)OnC1INH were used as control, then the pre-incubated proteins were added to mannan-coated ELISA plates in TBS-Ca^2+^ (2 mM CaCl_2_) at 25°C for 2 h. Among them, the OnMAp34, (r)OnMAp44, (r)OnMASP-1, (r)OnC1INH, and Trx were conjugated to biotin hydrazide (Sigma, USA), respectively. After incubation and three washings, the plate was added and incubated with streptavidin-HRP conjugate (1:2,500, Southern Biotech, USA) for 1 h at 37°C. At last, the reaction was detected at O.D. 450 by a microplate reader. Moreover, The interaction of (r)OnMAp34 and OnMBL was further detected by Far-western blot. Briefly, the (r)OnMAp34 and (r)OnMASP-1 proteins were incubated with biotin-labeled OnMBL (simultaneously adding 10 μg/mL mannan and 2 mM CaCl_2_) after SDS-PAGE, membrane transfer, and blocking. After incubation and three washings, the interaction of proteins was detected by streptavidin-HRP conjugate.

### Competitive Binding of OnMAp34 and OnMASP-1 to OnMBL

To investigate whether MBL and MAp34 binding sites were the same as MASP binding sites, competitive ELISA was performed. Briefly, fixed concentrations of (r)OnMASP-1 (2 μg/mL) and OnMBL (2 μg/mL) were incubated with increasing concentrations (0, 0.5, 2, 5, 10, 20, 40, 80, and 100 μg/mL) of (r)OnMAp34, (r)OnMAp44, and Trx, respectively; then the pre-incubated proteins were added to the mannan (10 μg/mL)-coated plates. Subsequently, the amounts of (r)OnMAp34 and (r)OnMAp44 binding to OnMBL were examined with ELISA using the streptavidin-HRP conjugate (1:2,500). Color reaction and reading were as mentioned above.

### Effect of OnMAps on LP

To investigate the effect of OnMAps On LP, an ELISA assay was performed as reported previously (41). Briefly, (r)OnMAp34, (r)OnMAp44, (r)OnC1INH, or Trx was added to diluted Nile tilapia serum in an MBL-binding buffer (20 mM Tris-HCl, 10 mM CaCl_2_, 1 M NaCl, 0.05%, Triton X-100, 0.1% BAS, pH 7.4); then the mixtures were incubated in mannan-coated plates for 3 h to form lectin–MASP complexes. After four washings, human C3 was added and incubated at 25°C for 3 h. After incubation and washing, the C3 fragments were detected with the mouse anti-C3b monoclonal antibody (Sigma, USA) as described above. Moreover, the amounts of bound (r)OnMAp34, (r)OnMAp44, and (r)OnC1INH in the complexes *in situ* were also examined, respectively.

### Statistical Analysis

The experiments were implemented three times and the data were presented as mean ± standard deviation (SD) by SPSS 17.0. The statistical significance was determined by analysis of variance (ANOVA) followed by a two-tailed Student *t*-test. The *p* values are defined by letters (a, b, c) (*p* < 0.05) or asterisks (^*^p < 0.05, ^**^p < 0.01). The figures were made using the Sigma Plot 10.0 software.

## Result

### Sequence Analysis of OnMAp34

**Figure 1:**
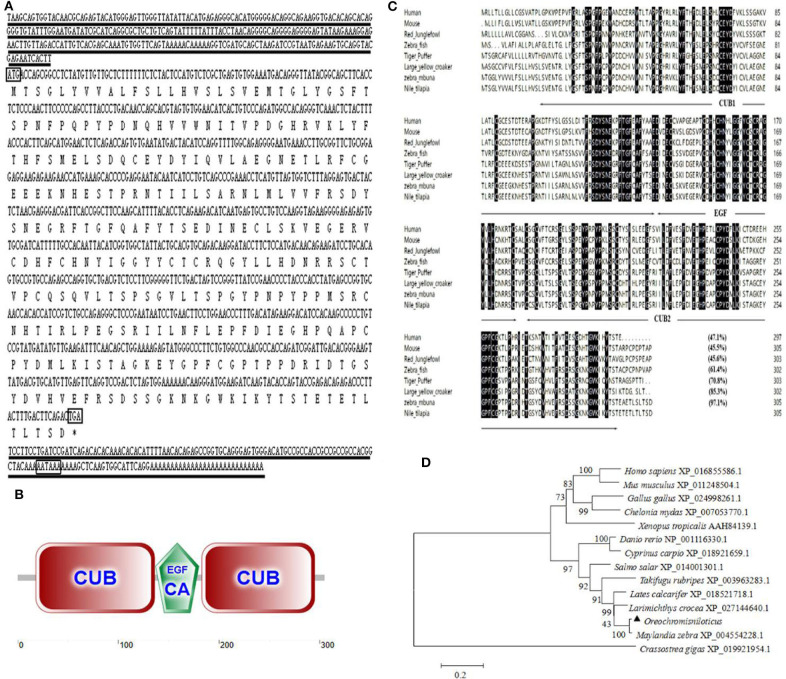
The full-length sequence and structural domains of OnMAp34. **(A)** The full length and deduced amino acid sequences of OnMAp34. The translation start, stop codons, and polyadenylation signal in the nucleotide sequences are in box. Asterisks indicate the terminate codes. The underlines represent the 5′-untranslated region (UTR) and 3′-untranslated region (UTR). **(B)** Structural domains of OnMAp34 predicted using SMART. The numbers represent the amino acid residues of OnMAp34. **(C)** Multiple-sequence alignment of the deduced amino acid sequence of MAp34 among different species. The conserved and identical residues are represented by black shading. The domain structure is indicated with arrows and lines spanning the appropriate length. **(D)** The phylogenetic tree of MAp34 family members was constructed using the NJ method by the MEGA 6 program based on the alignment of the MAp34s performed with the Clustal W method. Numbers at each branch indicated the percentage bootstrap values on 1,000 replicates.

An alternative splice product of *OnMASP-2* was cloned from Nile tilapia liver by PCR and was named mannose-binding lectin-associated protein OnMAp34 due to its predicted molecular mass of 34 kDa. The full length of *OnMAp34* was 1,352 bp, containing a 5′-untranslated region (UTR) of 281 bp and a 3′-UTR of 153 bp (Figure 1A). The 3′-UTR had a polyadenylation signal AATAAA and a polyadenylation tail. The ORF of *OnMAp34* was 918 bp encoding a 305-amino acid (aa) residue (Figure 1A). The structure domains of OnMAp34 encompassed a 119-aa CUB1, a 44-aa EGF, and a 113-aa CUB2 (Figure 1B). Moreover, the mature OnMAp34 protein had a theoretical isoelectric point of 5.41.

The amino acid of OnMAp34 shared relative high identity with *Homo sapiens* (47.1%), *Mus musculus* (45.5%), *Gallus gallus* (45.6%), *Danio rerio* (61.4%), *Takifugu rubripes* (70.8%), *Larimichthys crocea* (85.3%), and *Maylandia zebra* (97.1%) (Figure 1C). Moreover, phylogenetic tree analysis showed that MAp34s homology proteins from different species were distinctly classified into two clusters (Figure 1D). Among them, the OnMAp34 was closely related to the same family of *Maylandia zebra* but was very different from mammals, which was similar to the results of multiple sequence alignments. This reflected well the established phylogeny of chosen organisms and displayed the evolutionary relationships in accord with traditional taxonomy.

### Tissue Distribution of *OnMAp34*

**Figure 2:**
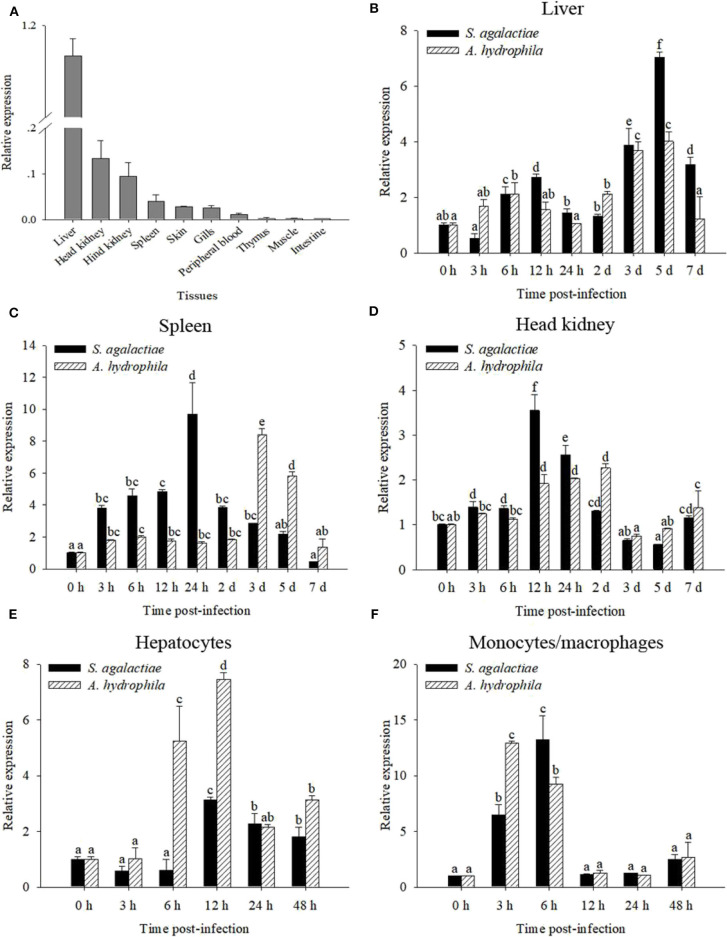
mRNA expression analysis of *OnMAp34* after pathogen infection. **(A)** Tissue distribution of *OnMAp34* mRNA in normal Nile tilapia. The ratio refers to the gene expression in different tissues relative to that in the liver and target gene expression is normalized against β-actin. Temporal mRNA expression of *OnMAp34* transcript in the liver **(B)**, spleen **(C)**, and head kidney **(D)** after *S. agalactiae* and *A. hydrophila* challenges. The mRNA expression of *OnMAp34* in the hepatocytes **(E)** and head kidney monocytes/macrophages **(F)** after *S. agalactiae* and *A. hydrophila* challenges. The error bars represent standard deviation (*n* = 9, three independent experiments and three individual fish per trial), and different letters (a, b, c, d, e, f) depict statistical significance between groups of tilapia after challenges and health (*p* < 0.05).

The transcript of *OnMAp34* was tested in many tissues from uninfected Nile tilapia (Figure 2A). The transcript of *OnMAp34* was at a high level in the liver and also expressed in the head kidney and hind kidney, whereas *OnMAp34* was at a low level in muscle and intestine. This result showed that OnMAp34 may be an important molecule and is widely distributed in various tissues.

### Expression Patterns of *OnMAp34* After Immune Challenge

To analyze the immune responses of *OnMAp34* upon pathogen infection in chosen tissues, qRT-PCR was implemented. The *OnMAp34* transcripts were significantly up-regulated within 5 d post-infection (p.i.) following *S. agalactiae* or *A. hydrophila* challenges in the liver (Figure 2B). In the spleen, the transcripts of *OnMAp34* were remarkably increased when the tilapia were challenged by *S. agalactiae* or *A. hydrophila* (Figure 2C). Additionally, the dynamic pattern of *OnMAp34* in the head kidney was similar to the spleen following pathogen infection, while *S. agalactiae* (9.7-fold) and *A. hydrophila* (8.4-fold) enhanced the levels of *OnMAp34* transcripts in the spleen to higher than those in head kidney (3.5- and 2.3-fold, respectively) (Figures 2C,D).

By isolating hepatocytes and MO/MΦ for *in vitro* stimulation experiments, the expression level of *OnMAp34* was detected (Figures 2E,F). In hepatocytes, the transcripts of *OnMAp34* were significantly unregulated within 12 h following *S. agalactiae* (3.1-fold) and *A. hydrophila* (7.4-fold) stimulation (Figure 2E). In MO/MΦ, *OnMAp34* was rapidly increased after *S. agalactiae* (6 h p.i.) and *A. hydrophila* (3 h p.i.) stimulation (Figure 2F).

### Expression and Purification of OnMAp34

**Figure 3:**
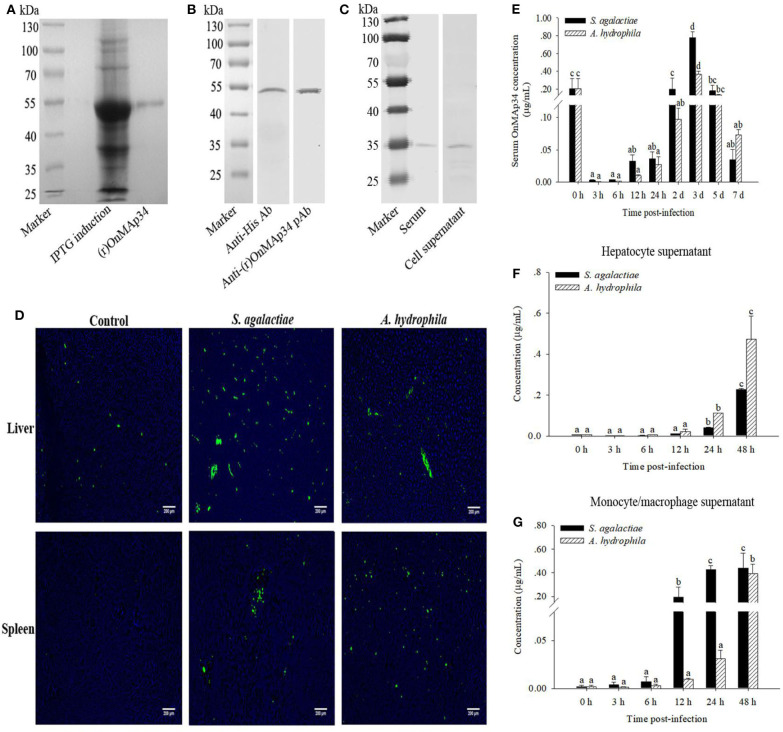
Protein expression analysis of OnMAp34 after pathogen infection. **(A)** Expression and purification of (r)OnMAp34. **(B)** Western blot analysis of (r)OnMAp34. **(C)** Western blot analysis of OnMAp34 in the serum and cell supernatant. The serum was collected at 3 d after *S. agalactiae* challenge and the supernatant was harvested from hepatocytes *in vitro* culture at 48 h after *A. hydrophila* stimulation. **(D)** OnMAp34 distribution revealed in the liver and spleen after *S. agalactiae* and *A. hydrophila* challenges by immunofluorescence microcopy. The liver at the time of 5 d after *S. agalactiae* or *A. hydrophila* stimulation; the spleen at the time of 24 h (*S. agalactiae*) or 3 d (*A. hydrophila*). Scale bar, 200 μm. **(E)** The dynamic change of serum OnMAp34 concentration after *S. agalactiae* and *A. hydrophila* stimulation. **(F,G)** The protein concentration of OnMAp34 in the supernatant of hepatocytes and head kidney monocytes/macrophages after *S. agalactiae* and *A. hydrophila* challenges. The error bars represent standard deviation (*n* = 9, three independent experiments and three individual fish per trial), and significant difference is indicated by different letters (a, b, c, d) (*p* < 0.05).

In Figure 3A, a single band (~54 kDa) was examined by SDS-PAGE analysis, which was consistent with the deduced size of (r)OnMAp34. To verify the specificity of pAb, the (r)OnMAp34 was detected by Western blot. The result revealed a positive and single band of about 54 kDa (Figure 3B). Moreover, a single band (~35 kDa) was detected in the serum and hepatocyte supernatant by Western blot (Figure 3C).

### Expression Dynamics of OnMAp34 *in vivo* and *in vitro*

The pAb was implemented to examine the OnMAp34 in the liver and spleen from tilapia after challenges of pathogens or PBS. As shown in Figure 3D, the expression of OnMAp34 (green fluorescent signal) in the liver revealed an amount of increase at 5 d p.i. In spleen, the fluorescence signal was not virtually examined in the control group, whereas the expression of OnMAp34 protein markedly increased after a challenge with *S. agalactiae* (24 h p.i.), and the similar up-regulation of OnMAp34 was also observed upon *A. hydrophila* (3 d p.i.) stimulation.

In the tilapia serum, the concentrations of OnMAp34 were significantly increased within 3 d after the challenges with *S. agalactiae* and *A. hydrophila*, and the peak reached at ~0.78 and 0.37 μg/mL, respectively (Figure 3E). In the cell supernatant, the expression levels of OnMAp34 were also remarkably up-regulated post infection (Figures 3F,G). Interestingly, the dynamic changes of OnMAp34 protein were similar to the *OnMAp34* transcripts after pathogen infection, but the peak expressions of OnMAp34 upon stimulations appeared later than those of *OnMAp34* in hepatocytes and MO/MΦ (Figures 3F,G).

### Effects of OnMAp34 on Inflammatory and Migration Reaction

**Figure 4:**
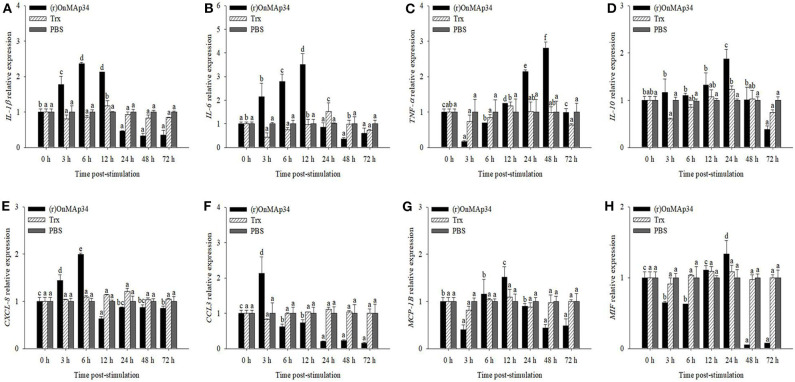
The mRNA expression of *IL-1*β **(A)**, *IL-6* **(B)**, *TNF-*α **(C)**, *IL-10* **(D)**, *CXCL-8* **(E)**, *CCL3* **(F)**, *MCP-1B* **(G)**, and *MIF* **(H)** from Nile tilapia in the monocytes/macrophages. Nile tilapia head kidney monocytes/macrophages were treated with (r)OnMAp34 (5 μg/mL), Trx (5 μg/mL), and PBS. The error bars represent standard deviation (*n* = 9, three independent experiments and three individual fish per trial) and significant difference is indicated by different letters (a, b, c, d, e, f) (*p* < 0.05).

To explore the inflammation and migration response upon the stimulation of OnMAp34, the present study analyzed the expression patterns of inflammatory cytokines and chemotactic factors in MO/MΦ after treatment with (r)OnMAp34 and Trx. After (r)OnMAp34 stimulation, the transcripts of inflammatory factors were rapidly and significantly increased, including *IL-1*β, *IL-6, TNF-*α, and *IL-10*. Among them, the transcripts of *IL-1*β and *IL-6* were enhanced rapidly with the increase of 2.4-fold at 6 h p.i. and 3.5-fold at 12 h p.i., respectively (Figures 4A,B), while the transcript of *IL-10* was enhanced with the increase of 1.9-fold at 24 h p.i. (Figure 4D). Moreover, the expression of *TNF-*α was significantly enhanced with the increase of 2.8-fold at 48 h p.i. (Figure 4C). According to the results of *CXCL-8, CCL3, MCP-1B*, and *MIF* after the stimulation of (r)OnMAp34, the expressions also markedly increased. However, the transcripts of *CXCL-8, CCL3*, and *MCP-1B* were rapidly increased at 6, 3 h, and 12 h p.i., respectively, while the *MIF* significantly up-regulated at 24 h p.i. (Figure 4).

### OnMAp34 Promotes Phagocytosis

**Figure 5:**
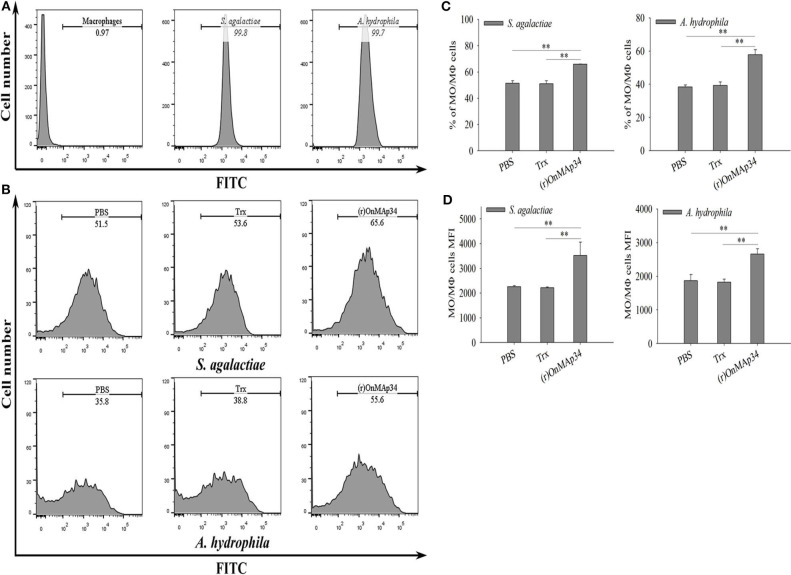
Effects of (r)OnMAp34 on phagocytosis of Nile tilapia monocytes/macrophages. Flow cytometric analysis of the monocytes/macrophages phagocytosing *S. agalactiae* and *A. hydrophila*. Data show analyses of 10,000 events. **(A)** The histogram of the cells alone, *S. agalactiae* alone and *A. hydrophila* alone. The marker represented phagocytosis part. **(B)** The histogram of flow cytometric analyses of the monocytes/macrophages phagocytosing *S. agalactiae* and *A. hydrophila* pre-incubated with PBS, Trx, or (r)OnMAp34. The phagocytosis rates were shown near the marker. The results shown here were from one representative experiment out of three independent experiments. **(C,D)** The total average % (phagocytic index) and the mean fluorescence intensity (MFI) of monocytes/macrophages. The average standard deviation was obtained from three independent experiments. The symbol * shows a significant difference from control (*p < 0.05, **p < 0.01).

To investigate the mechanism of OnMAp34 promoting bacterial clearance, the bacterial phagocytosis of MO/MΦ was performed by FCM. As shown in Figure 5A, the *S. agalactiae* and *A. hydrophila* were successfully labeled by FITC, and MΦ alone did not show any fluorescence. The (r)OnMAp34 could significantly enhance the phagocytosis of MO/MΦ against *S. agalactiae* and *A. hydrophila* (Figure 5B). The phagocytic percentage (Figure 5C) and phagocytic index (Figure 5D) of the (r)OnMAp34 group were significantly higher than those in the control group.

### OnMAp34-Mediated Inhibition of OnMASP-1 Binding to OnMBL

**Figure 6:**
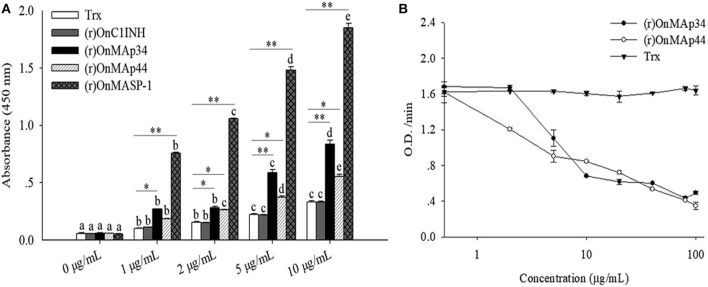
OnMAp34 mediated inhibition of OnMASP-1 binding to OnMBL. **(A)** Binding of OnMAps and OnMASP-1 to OnMBL. Protein-protein interaction was determined by ELISA. Briefly, OnMBL was incubated with increasing concentrations of (r)OnMAp34, (r)OnMAp44, or (r)OnMASP-1, respectively. The Trx and (r)OnC1INH were used as control. The mixtures were then incubated in mannan-coated microtiter wells, then the bound OnMASPs were detected with streptavidin-HRP Ab. **(B)** OnMAp34 could compete to inhibit the binding of OnMASP-1 and OnMBL. Fixed concentrations of (r)OnMASP1-HA (labeled with biotin hydrazide) and OnMBL were incubated with various amounts of (r)OnMAp34, (r)OnMAp44 or Trx. After incubation, the MBL-containing complexes were captured in microtiter wells coated with mannan, then the bound (r)OnMASP1 was detected with streptavidin-HRP Ab. The error bars represent standard deviation (*n* = 4). The significant difference of the same protein at different time points is indicated by different letters (a, b, c, d, e) (*p* < 0.05), and the significant difference between different protein groups at the same time point is indicated by asterisks (*p < 0.05, **p < 0.01).

To explore the combination of the OnMAp34 and OnMASP-1 with OnMBL, the interaction pattern of protein was detected by ELISA assay. The results indicated that the (r)OnMAp34, (r)OnMAp44, and (r)OnMASP-1 could bind to the OnMBL in a concentration-dependent manner (Figure 6A). By contrast, (r)OnC1INH and Trx could not bind to OnMBL (Figure 6A). Moreover, the (r)OnMAp34 and (r)OnMASP-1 could also bind to the OnMBL by Far-western blot analysis (Figure S2). Since MAps have the same binding domains as MASPs, in order to determine whether MAps compete with MASPs for binding to MBL, the ELISA assay was performed. Using the competition ELISA assay, We found that the binding of (r)OnMASP-1 reduced with a dose-dependent manner by adding plenty of (r)OnMAp34 and (r)OnMAp44 (Figure 6B). This result suggested that the OnMAp34, OnMAp44, and OnMASP-1 bind to the same or overlapping sites on the OnMBL.

### OnMAps Down-Regulates MASP-Mediated LP

**Figure 7:**
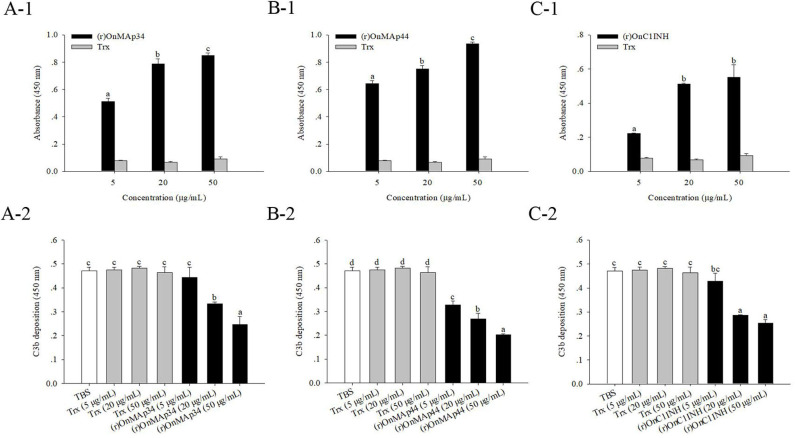
OnMAp34 mediated inhibition of complement C3 binding. **(A)** (r)OnMAp34 or Trx was pre-incubated with the Nile tilapia serum, then the complexes were allowed to bind to a mannan-coated microtiter well. Followed by incubation with human C3, the C3 fragments were finally detected by ELISA. Moreover, the amount of bound (r)OnMAp34 in the complexes *in situ* was also detected by ELISA. The (r)OnMAp44 **(B)** and (r)OnC1INH **(C)** were used as control. The error bars represent standard deviation (*n* = 4), and significant difference is indicated by different letters (a, b, c, d) (*p* < 0.05).

Combining the above results of OnMAp34 competition with OnMASP-1 for binding to OnMBL, the effect of OnMAp34 on MASP-mediated LP was explored. As shown in Figure 7, the (r)OnMAp34 or (r)OnMAp44 was incubated with the Nile tilapia serum, then the number of C3 fragments reduced with the addition of OnMAps in a dose-dependent manner, whereas Trx did not decrease the number of C3 fragments (Figures 7A,B). Moreover, the conventional inhibitory protein OnC1INH in MASP-mediated LP could reduce the amount of C3 fragments (Figure 7C). Therefore, we speculated that MAp34 competes for the binding to MBL in Nile tilapia, leading to a reduction of MASPs binding to MBL, inhibiting the MASP-mediated LP of complement activation.

## Discussion

The LP of the complement system is one of the main components of innate immunity and plays a pivotal role in host defense against invading microorganisms (8). MASPs are the important constituents of the LP, which play indispensable roles in the activation and regulation of the proteolytic cascade reaction (7, 8). MAp34, like MAp19, is a novel alternative splice product of the *MASP-2* gene. At present, the effect function and related mechanism of MAp34 in the host innate immune system remains unknown, such as the lectin complement pathway and non-specific cell immunity. In this study, we identified and functionally characterized homolog *MAp34* for the first time in *O. niloticus*. Intriguingly, the OnMAp34 functions as an inhibitor of the lectin pathway via competitive inhibition of OnMASPs activity. Moreover, OnMAp34 also participates in the non-specific cellular immune defense, including the regulation of inflammation, migration, and enhancement phagocytosis of monocytes/macrophages.

MAp34 is a 34-kDa non-enzymatic protein produced by the alternative splicing of the *MASP-2* gene, which only contains three domains (CUB1-EGF-CUB2). The CUB1-EGF-CUB2 domains are responsible for the Ca^2+^-dependent association of MASPs dimers with MBL or ficolins, while the CCP and SP domains are involved in interactions and catalysis with complement molecules (C2, C4, C3), respectively (8). OnMAp34 lacks the two CCP domains and the entire SP domain, which has no catalytic function and is likely to be unable to interact with complement factors. However, the OnMAp34 may involve in interaction with the collagen-like region (CLR) of MBL and FCNs, which plays pivotal roles in the regulation of LP (8, 17). The 3′-UTR of OnMAp34 has a typical polyadenylation signal (AATAAA), like the OnMASP-1 and OnMAp44 (25, 29). Moreover, OnMAp34 contains a 19-aa signal peptide and has no transmembrane domain, indicating that it is likely to function primarily in the extracellular environment. In addition, the domain of MAp34 is highly conserved. We speculated that OnMAp34 may have the similar function at that in other species.

Tissue distribution analysis showed that *OnMAp34* was predominantly expressed in the liver and widely exists in other tissues. The result implied that the liver may be the major organ to produce MAp34, which was similar to the finding in MASP-1, MASP-2, and MAp19 of humans (8, 42). After pathogen infection, the expression of *OnMAp34* was remarkably enhanced in the liver, which was consistent with other MASPs. For example, in grass carp, the *gcMASP-1* transcripts were markedly increased in the liver post infection with *A. hydrophila* (27). The expression of *OnMAp44* in the liver from Nile tilapia was rapidly enhanced after pathogen infection (25). This result implied that the liver may be an important organ, contributing significant expression of MASPs in host defense against pathogen invasion, including MAp34. In teleost fish, the spleen and head kidney are very important immune organs that play essential roles in the immune response (43–45). The expression levels of *OnMAp34* in spleen and head kidney were significantly increased upon challenges with *S. agalactiae* and *A. hydrophila*, which were similar to other MASPs, such as MASP-1 and MAp44 (25, 27, 29). Further research showed that the mRNA and protein expression levels of OnMAp34 in hepatocytes and head kidney MO/MΦ were significantly up-regulated following infection with the pathogen. Moreover, OnMAp34 was remarkably increased in tilapia serum by pathogen infection. Taken together, these results indicated that OnMAp34 is induced by bacterial challenges, which is likely to participate in host defense against bacterial infection. The information of OnMAp34 in different tissues and its response to immune stimulus are important in understanding their roles in innate immunity.

In humans, studies have found that MASPs can participate in inflammatory response (7, 8). The recombinant MASP-1 was able to induce the release of IL-6 and IL-8 in human endothelial cells and the chemotaxis of neutrophil granulocytes (46). A high concentration of MASP-2 easily promotes inflammation and proliferation, leading to tumor invasion and metastasis (47). MAp44 is also considered to be a potentially available therapy of inflammatory arthritis (24). In this study, the expressions of the cytokines (*IL-1*β, *IL-6, TNF-*α, and *IL-10*) and chemokines (*CXCL-8, CCL3, MCP-1B*, and *MIF*) from MO/MΦ were rapidly and significantly up-regulated after (r)OnMAp34 stimulation, which was similar to the findings in MASP-1 and MAp44 from Nile tilapia (25, 29). Recombinant MAp34 from *Cynoglossus semilaevis* could also activate peripheral blood lymphocytes and promote the production of inflammatory cytokines, including IL-1β, IL-6, IL-8, and TNF-α (30). Moreover, we found that OnMAp34 could enhance the phagocytosis of pathogens by MO/MΦ. As we know, MO/MΦ are important effector cells that eliminate pathogenic microorganisms, which respond quickly to invading microorganisms and exert a pivotal role in non-specific cellular defense. The non-specific cellular immune defense is an important component of the fish innate defense system, including inflammation, migration, phagocytosis, and killing (37, 48). OnMAP34 could not only involve the regulation of inflammation and migration reaction but also enhance MO/MΦ-mediated phagocytosis, thereby playing a regulatory role in host non-specific cellular defense. Therefore, we speculate that the non-enzyme protein MAP34 is likely to exert a role similar to opsonins in non-specific cellular defense.

In mammals, MBL recognizes the target molecule and interacts with the CUB1-EGF-CUB2 domain of activated MASPs through its CLR to form MBL-MASP complexes, thereby initiating a series of cascade enzymatic reactions (7, 8, 49). Recombinant MAp44 in human could compete with MASP-2 or MASP-1 for binding to MBL and FCN (50, 51). Compared with MASP-2, MASP-1, MASP-3, and MAp44, MAp19 has more than 10-fold lower affinity for MBL, which may be caused by the absence of the CUB2 domain (42). In this study, we found that OnMAp34 was able to bind significantly to OnMBL and also compete with OnMASP-1 for binding to OnMBL. This result suggested that the OnMAp34 and other OnMASPs were likely to bind to the same or overlapping sites on the OnMBL. Noticeably, the OnC1INH could not be combined with OnMBL. As an important serine protease inhibitor, C1INH exerts a role in the regulation of lectin pathway by interacting with activated MASP-1 and MASP-2 (20, 52). However, OnMAp34 and OnMAp44 could inhibit the lectin-activated complement pathway by competing with OnMASPs for binding to OnMBL, which was consistent with the results of MAp44 in humans (16, 17, 50). Resent research has shown that MASP-1 and MASP-2 are indispensable in LP activation in humans (7, 23). In human serum, MASP2 activation depends on MASP1, and inhibition of MASP-1 can permanently block the MASP-2 activity (4, 23). Activated MASP-2 recognizes C4 or C2 through its two CCP domains and catalyzes the SP domain to cleave complement factors to form a C3 convertase, which in turn cleaves C3 to initiate a subsequent series of cascade enzymatic reactions (7, 8). Further, C3 is the central role in the complement system and is indispensable for the activation of this system (53). Therefore, the blocking of the C3 cleavage can completely abolish the activation of the complement system. In the current study, OnMAP34 could not recognize related serum complement factors without a CCP domain, and only compete with other OnMASPs to bind OnMBL, which led to the blocking of downstream enzymatic cascades and thus potent-inhibited the lectin-activated complement pathway.

**Figure 8:**
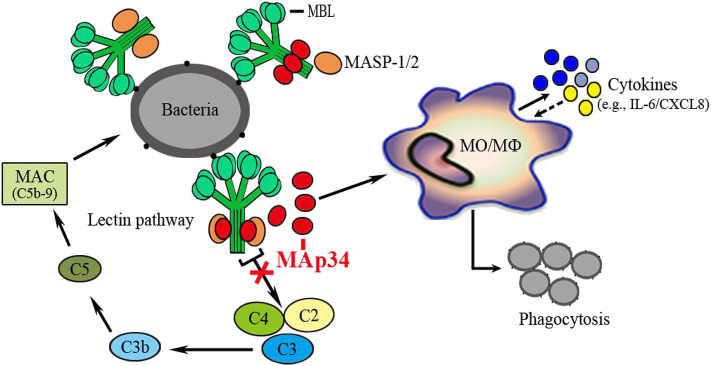
OnMAp34 regulates the non-specific cell immunity of monocytes/macrophages and inhibits the lectin pathway of complement activation in teleost fish. A proposed model to illustrate the novel mechanism of OnMAp34 as a potent regulator in the lectin complement pathway and non-specific cell immunity. The OnMAp34 could compete with OnMASPs to combine OnMBL and inhibit the lectin pathway of complement activation. It also participated in the non-specific cellular immune defense, including the regulation of inflammation, migration, and enhancement phagocytosis of monocytes/macrophages. This provided innate immune regulation against infection.

In conclusion, the present study demonstrated an evolutionarily conserved regulator of the lectin complement pathway in an early vertebrate, as schematically illustrated in Figure 8. A MAp34 gene (OnMAp34) was identified from *O. niloticus*. After bacterial infection, the mRNA and protein expressions of OnMAp34 showed significant up-regulation *in vivo* and *in vitro*. Further, we found that OnMAp34 could participate in non-specific cellular immune defense, including the regulation of inflammation, migration, and enhancement of the phagocytosis of monocytes/macrophages. Moreover, OnMAp34 functions as a potent inhibitor of the lectin complement pathway via competitive inhibition of OnMASPs activity. Overall, our results provide new insights into the understanding of MAp34 as an important regulator in the lectin complement pathway and non-specific cell immunity in an early vertebrate.

## Data Availability Statement

All datasets presented in this study are included in the article/Supplementary Material.

## Ethics Statement

The animal study was reviewed and approved by University Animal Care and Use Committee of the South China Normal University.

## Author Contributions

JY and LM conceived and designed the experiments. LM and XY performed the experiments and analyzed the data. LM wrote the manuscript. HW and KH performed Nile tilapia farming and samples collection. ZG and JY reviewed the manuscript. All authors contributed to the article and approved the submitted version.

## Conflict of Interest

The authors declare that the research was conducted in the absence of any commercial or financial relationships that could be construed as a potential conflict of interest.
