# Supplementary material for: MAp34 Regulates the Non-specific Cell Immunity of Monocytes/Macrophages and Inhibits the Lectin Pathway of Complement Activation in a Teleost Fish
Source: Front Immunol. 2020 Aug 12;11:1706. doi: 10.3389/fimmu.2020.01706 (PMC7435015; doi:10.3389/fimmu.2020.01706)

**Supplemental material**

**Fig. S1.** OnMAp34 distribution revealed in liver and spleen after *S. agalactiae* and *A. hydrophila* challenges by immunofluorescence microcopy. The liver at the time of 5 d after *S. agalactiae* or *A. hydrophila* stimulation; and the spleen at the time of 24 h (*S. agalactiae*) or 3 d (*A. hydrophila*). The primary antibody was the mouse serum (no OnMAp34 protein immunization), the fluorescent antibody was goat-anti-mouse IgG Alexa 488 (Thermo, USA). Scale bar, 200 μm.


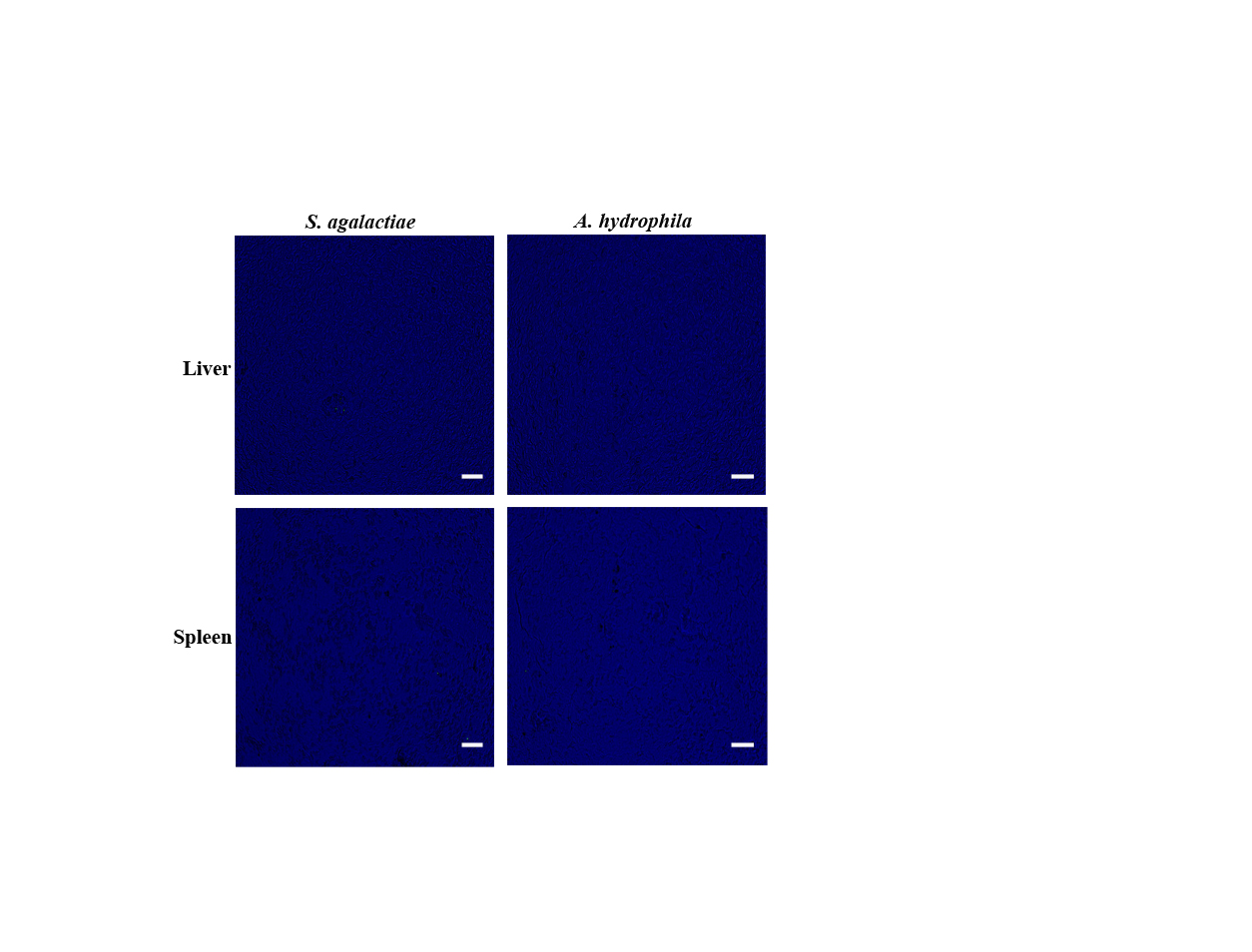


**Fig. S2.** Binding of OnMAp34 and OnMASP-1 to OnMBL by Far-western blot. (**A**) SDS-PAGE detection of purified protein (unreduced). (**B**) The (r)OnMAp34 and (r)OnMASP-1 proteins were incubated with biotin-labeled OnMBL after SDS-PAGE, membrane transfer, and blocking. Then the interaction of proteins was detected by streptavidin-HRP conjugate. Lane 1, marker; Lane 2, (r)OnMASP-1; Lane 3, (r)OnMAp34; Lane 4, (r)OnC1INH.


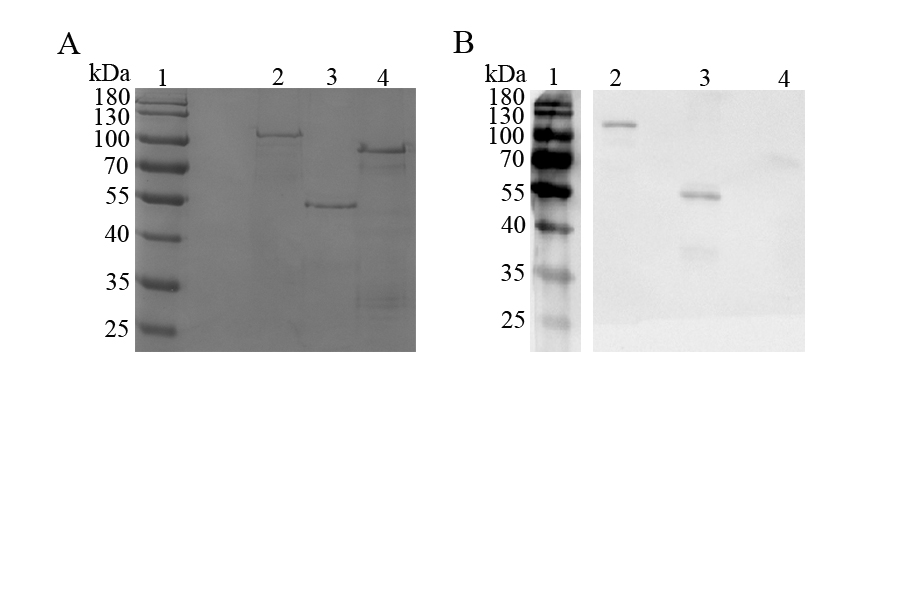

Supplement: Supplementary file 1 [file Data_Sheet_1.doc]
